# Exploration of Pedestrian Head Injuries—Collision Parameter Relationships through a Combination of Retrospective Analysis and Finite Element Method

**DOI:** 10.3390/ijerph13121250

**Published:** 2016-12-16

**Authors:** Wenjun Liu, Sen Su, Jinlong Qiu, Yongyong Zhang, Zhiyong Yin

**Affiliations:** 1 Institute for Traffic Medicine, Department 4th, Institute of Surgery Research, Daping Hospital, Third Military Medical University, Chongqing 400042, China; liuwenjun@dphospital.tmmu.edu.cn (W.L.); 1441suse@163.com (S.S.); tmmu_c@foxmail.com (J.Q.); 2 Transit College, Chongqing Jiotong University, Chongqing 400074, China; wowcszy@cqjtu.edu.cn

**Keywords:** car–pedestrian collision, head injuries, retrospective analysis, FEM

## Abstract

There are a very limited number of reports concerning the relationship between pedestrian head injuries and collision parameters through a combination of statistical analysis methods and finite element method (FEM). This study aims to explore the characteristics of pedestrian head injuries in car–pedestrian collisions at different parameters by using the two means above. A retrospective analysis of pedestrian head injuries was performed based on detailed investigation data of 61 car–pedestrian collision cases. The head damage assessment parameters (head injury criterion (HIC), peak stress on the skull, maximal principal strain for the brain) in car–pedestrian simulation experiments with four contact angles and three impact velocities were obtained by FEM. The characteristics of the pedestrian head injuries were discussed by comparing and analyzing the statistical analysis results and finite element analysis results. The statistical analysis results demonstrated a significant difference in skull fractures, contusion and laceration of brain and head injuries on the abbreviated injury scale (AIS)3+ was found at different velocities (*p* < 0.05) and angles (*p* < 0.05). The simulation results showed that, in pedestrian head-to-hood impacts, the values of head damage assessment parameters increased with impact velocities. At the same velocity, these values from the impact on the pedestrian’s back were successively greater than on the front or the side. Furthermore, head injury reconstruction and prediction results of two selected cases were consistent with the real injuries. Overall, it was further spelled out that, for shorter stature pedestrians, increased head impact velocity results in greater head injury severity in car–pedestrian collision, especially in pedestrian head-to-hood impacts. Under a back impact, the head has also been found to be at greater damage risk for shorter stature pedestrians, which may have implications on automotive design and pedestrian protection research if prevention and treatment of these injuries is to be prioritized over head injuries under a front or side impact.

## 1. Introduction

In car–pedestrian collisions, the pedestrian’s head can suffer from multiple injuries under different load conditions, such as skull fractures and brain injuries with different distributions and severities. Reducing pedestrian head injury risk and guiding a better diagnosis and treatment for head traffic injuries requires a clear understanding of the collision events and head injuries. In recent decades, corpse and dummy experiments and the mathematical dynamic model (MADYMO) are the main methods for studying the responses and damage mechanisms of the head in vehicle crashes. Due to the ethical and legal issues, as well as the poor reproducibility, corpse research is carried out less often. Crash tests with physical dummies are widely used in auto companies, and it greatly boosted the research of biomechanics and automotive safety technology. MADYMO is frequently employed for kinematic predictions [1,2,3], while MADYMO pedestrian models are of a simple structure without a complex variety of materials and properties. Therefore, physical dummies and MADYMO pedestrian models had shortcomings in evaluation of fractures and internal injuries [4,5,6]. The biomechanical dummy model established by finite element method (FEM) can not only reflect the complex anatomy of the human body, but also accurately describe the dynamic response of the body; therefore, it is widely used in research of human biomechanics and injury mechanisms [7,8,9].

Finite-element (FE) human body models (HBMs) such as the H-model, Ford Human Body Model, Wayne State University (WSU) Human Model, Global Human Body Models Consortium (GHBMC) model, Human Model for Safety (HUMOS)2, and Total Human Model for Safety (THUMS) have been widely used in the injury biomechanics field [10]. Mordaka [11] evaluated the influence of head rotational kinematics on head injury and injury criteria using the FE head and windshield models. Watanabe [12] investigated the influence of vehicle body type, pedestrian body size and impact location as well as the collision speed on pedestrian injury, using three different vehicle FE models and three different pedestrian FE models (THUMS, version 4.0). By using THUMS (version 1.4), Alvarez [13] investigated the influence of neck muscle activation in a simulation of a pedestrian accident, and addressed the effect of muscle activation on strain in the brain, when initiated at the time of head contact. Fahlstedt [14] compared and evaluated the difference in head kinematics between the Netherlands Organization for Applied Scientific Research (TNO) and THUMS (version 1.4) models in pedestrian accident situations. It was found that, although there were differences in kinematics between the THUMS and TNO pedestrian models, the two models showed similar trends for the head trajectory when various parameters were altered.

Car–pedestrian impact parameters mainly include contact angle and impact speed. The role of speed in pedestrian crashes has been confirmed through numerous studies. For example, Nilsson [15], and Elvik [16], demonstrated that lower mean traffic speeds in response to speed limit reduction result in reduced likelihood of fatal crashes. Much of this research points to the fact that even small reductions in speed can lead to considerable reductions in pedestrian trauma. Severity of crash outcomes in response to speed have also been well researched. Other studies showed that fatal crashes decline more substantially with the same amount of mean speed reduction than all injury crashes [17,18,19]. Jurewicz [18] concluded that pedestrian–vehicle impact speeds of approximately 20 km/h were likely to produce Maximum Abbreviated Injury Scale (MAIS)3+ injury probability of approximately 10%, after reviewing available international research on relationships between impact velocity change (Δv), impact speeds, and the probability of fatal and serious injury (MAIS3+) across a range of common crash scenarios.

Elliott [19] used the MADYMO MultiBody (MB) pedestrian model to quantitatively analyze the influences of pedestrian speed and pedestrian gait on the transverse translation of the pedestrian’s head, head rotation about the vertical head axis, and head impact velocity. Head rotation is related to pedestrian stance at impact, which is known to affect the kinematics of a collision. Richards [20] explored the relationship between age and the different types of head injury received by pedestrians in traffic accidents with cars. The principle result was that the risk of intracranial injury increased with age, whilst the risk of fracture to the head or facial bones remained relatively constant.

Although numerous studies regarding vehicle–pedestrian crashes have been performed to prevent pedestrian injuries from collisions in developed countries as well as some developing countries [21,22,23,24,25,26,27,28,29,30], it is of particular importance to address some characteristics concerning the relationship between collision parameters and pedestrian injuries, especially in developing countries, such as China. This study explores the characteristics of pedestrian head injuries in car–pedestrian collisions under different parameters by using a statistical analysis method and FEM, which could provide a theoretical basis for automotive design and prevention and treatment of pedestrian injuries.

## 2. Materials and Methods

### 2.1. Selection and Analysis of Injury Data

A retrospective analysis of pedestrian head injuries from car-pedestrian collisions from 2010 to 2014 in Chongqing, China, was performed. Sixty-one cases with detailed injuries and determined impact parameters were selected to diagnose and analyze the pedestrian head injuries according to 'The Abbreviated Injury Scale 2005 (AIS 2005) Revision’ by the Association for the Advanced of Automotive Medicine [31], from AIS 1 (minor), AIS 2 (moderate), AIS 3 (serious), AIS 4 (severe), AIS 5 (critical), to AIS 6 (currently untreatable). In these 61 casualties, there were 38 males and 23 females, with a mean age of 51 and mean height of 154 cm. Of the head injuries, AIS 5 accounted for 14/61, AIS 4 for 24/61, AIS 3 for 16/61, AIS 2 for 5/61, and AIS 1 for 2/61. Since there was a lack of cases under front impact, the contact angles were classified as left, right, and back. The car impact speed was calculated from the braking distance or the pedestrian throwing distance [32], or it was obtained from videos [33] or an event data recorder (EDR) [34]. Cases were grouped into three categories according to impact speed: 25–39, 40–55, and ≥55 km/h. After the data was collected and consolidated, data rates were compared by a Fisher’s Exact Test, taking *p* < 0.05 as significant.

### 2.2. Finite Element Model of Car

**Figure 1:**
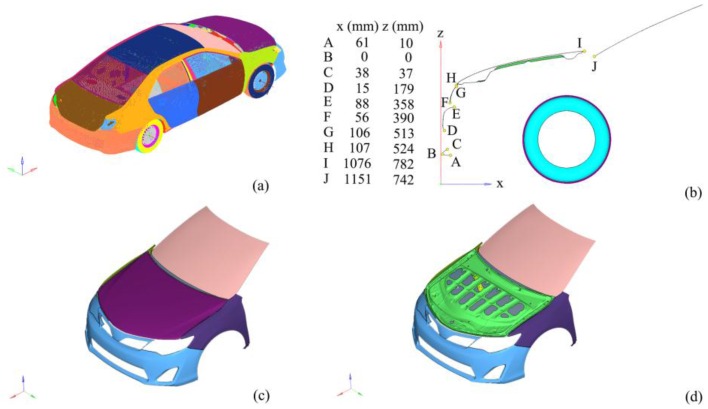
Vehicle front structure. (**a**) Vehicle outline drawing; (**b**) Vehicle centerline geometry; (**c**) Engine hood outer panel; (**d**) Engine hood inner panel.

The vehicle model shown in Figure 1 was chosen from the National Car Crash Analysis Center (NCAC [35,36,37,38]), which was validated as the car model for finite element analysis [9,39]. The dimensions of this model are approximately 4.80 × 1.82 × 1.44 m. It has more than 1.67 million grid cells, and the average element size is 6–7 mm. The material and property definition of this model met the basic regulatory requirements for vehicle dynamics simulation studies.

### 2.3. Finite Element Model of Pedestrian Head

**Figure 2:**
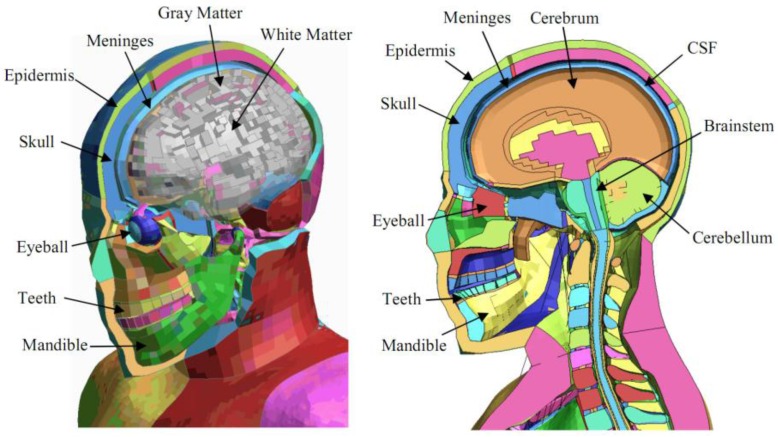
Head finite element model [40,42].

The THUMS (version 4.0, standing posture) finite element model which was developed, designed, and verified by Toyota Motor Corporation and Toyota Technical Center (Nagakute, near Nagoya in Aichi, Japan) was used as the pedestrian finite element model [12,40]. This model simulates a 50th percentile American male with a height of 175 cm and a weight of 77 kg, and it has been validated for pedestrian impacts [40,41]. THUMS 4.0 includes the internal organs of the body, the brain, and the skeleton, in great detail. The number of nodes in the model is approximately 650,000 and the number of elements is approximately two million. The model used a high resolution computed tomography (CT) scanner to scan the human body, get the body structure parameters, and then build human digital models which accurately reflected human anatomical features [9,40,41]. As shown in Figure 2, for the THUMS (version 4.0, standing posture), the head model includes the epidermis, skull, mandible, eyeballs, teeth, meninges, cerebrum, cerebellum, cerebrospinal fluid (CSF), etc. The meninges part has three layers: dura mater, arachnoid, and pia mater. The brain parts include the white matter and the gray matter.

### 2.4. Crash Simulation Design and Injury Assessment Indicators

**Figure 3:**
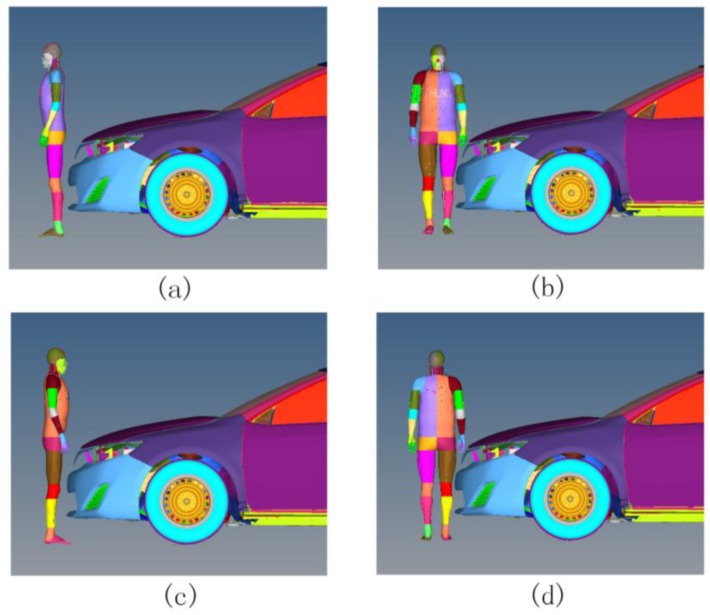
Car–pedestrian collision simulations at different contact angles. (**a**) Collision on the back of the head; (**b**) Collision on the left side of the head; (**c**) Collision on the front of the head; (**d**) Collision on the right side of the head.

Studies show that 95% of pedestrian collisions occurred at the impact speed of no more than 60 km/h, and most were in the range of 25–55 km/h, often resulting in severe damage [9,43]. Therefore, the car collision velocities in this study were set at less than 60 km/h, which were 25, 40 and 55 km/h, with selections of the contact angles on the pedestrian’s back, left side, front and right side. The contact parts between the pedestrian and the car in this study are located in the centerline area of the vehicle’s front structure. Surface-to-surface contact was chosen as the contact type, and the dynamic friction coefficient was set as 0.65. The hypermesh pre-process tool was used to build simulations of the car-pedestrian collisions at different contact angles, as shown in Figure 3. In addition, differences in physical status such as height and weight could lead to changes of pedestrian head–vehicle impact types and head injury severity [44]. Therefore, a model scaling process was adopted to the pedestrian model by hypermesh. The most accurate head impact conditions could be achieved when THUMS was scaled with one z-factor adjusting its height and one x-y-factor adjusting its weight. According to the accident investigation data, the average height of pedestrians used in retrospective analyses was 154 cm, and the corresponding standard weight for the Chinese population was 48.6 kg. Thus, the THUMS height scaling factor was identified as 0.87, and the THUMS mass scaling factor was identified as 0.63. LS-DYNA was used to calculate the collision process.

The head injury criterion (HIC) is a measure of the likelihood of a head injury arising from an impact. At an HIC of 1000, there is an 18% probability of a severe head injury, a 55% probability of a serious injury and a 90% probability of a moderate head injury to the average adult [45]. The stress in the skull bone has been proven to be a predictor of the risk of skull fractures, and the maximum stress of an adult skull was 96.53 MPa [5,46]. The stress from the compact bone of the skull was obtained by the post-process tool hyperview in this study. Research suggests that distortional strain could be used to indicate the risk of traumatic brain injury, since the brain tissue can be considered as a fluid in the sense that its primary mode of deformation is shear [47,48]. The maximal principal strain obtained by hyperview was chosen as the predictor of brain injuries. It is suggested that a maximal principal strain higher than 0.18–0.21 leads to diffuse axonal injury (DAI) while vascular rupture is expected at strain levels above 0.30–0.60 [11].

### 2.5. Cases Descriptions

Two cases of adult pedestrians were chosen from the 61 cases described above and simulated using the THUMS 4.0 and FE car model. The initial conditions and the descriptions for the selected cases can be seen in Table 1. Real injury and reconstruction results are discussed in sections below.

## 3. Results

### 3.1. Statistical Analysis of Pedestrian Head Injuries

In the 61 victims of the car–pedestrian collisions, there was a significant difference in skull fractures, contusion and laceration of the brain, and head injuries AIS3+ at different collision velocities (*p* < 0.05, Table 2). At the impact speed of 25–39 km/h, the common forms of pedestrian head injuries were unilateral contusion and laceration of the scalp (8/12). The majority did not have skull fractures (9/12) or contusions and lacerations of the brain (11/12), and the proportion of head injuries at AIS3+ was 7/12. At collision velocities of 40–55 km/h, the common forms of pedestrian head injuries were unilateral contusion and laceration of scalp (11/21) and unilateral skull fractures (11/21). The majority did not have contusions and lacerations of the brain (19/21) and the proportion of head injuries at AIS3+ was 19/21. At ≥55 km/h, there were unilateral contusion and laceration of scalp (14/28), unilateral skull fractures (19/28), without contusion and laceration of brain (14/28), and the proportion of head injuries at AIS3+ was 28/28.

The results presented in Table 3 demonstrated that a significant difference for pedestrian head injuries was found at different contact angles (*p* < 0.05). When the left side of the pedestrians’ heads were impacted by the car, it often caused skull fractures on the left side (19/33), the proportion of contusions and lacerations of the scalp on the left side (2/33) was lower than that on the right side (18/33), the proportion of contusions and lacerations of the brain on the left side (5/33) was higher than that on the right side (0/33), and the proportion of head injuries at AIS3+ was 28/33. When the right side of the pedestrians’ heads were impacted by the car, it often resulted in skull fractures on the right side (11/21), the proportion of contusions and lacerations of the scalp on the right side (4/21) was lower than that on the left side (8/21), the proportion of contusions and lacerations of the brain on the right side (3/21) was higher than that on the left side (0/21), and the proportion of head injuries at AIS3+ was 19/21. When the pedestrians’ heads suffered a back impact, skull fractures, contusions and lacerations of the scalp, and contusions and lacerations of the brain often occurred bilaterally, and the proportions were 4/7, 4/7, and 2/7, respectively. In addition, the proportion of head injuries at AIS3+ was 7/7.

### 3.2. Finite Element Analysis of Pedestrian Head Injuries

#### 3.2.1. HIC

**Figure 4:**
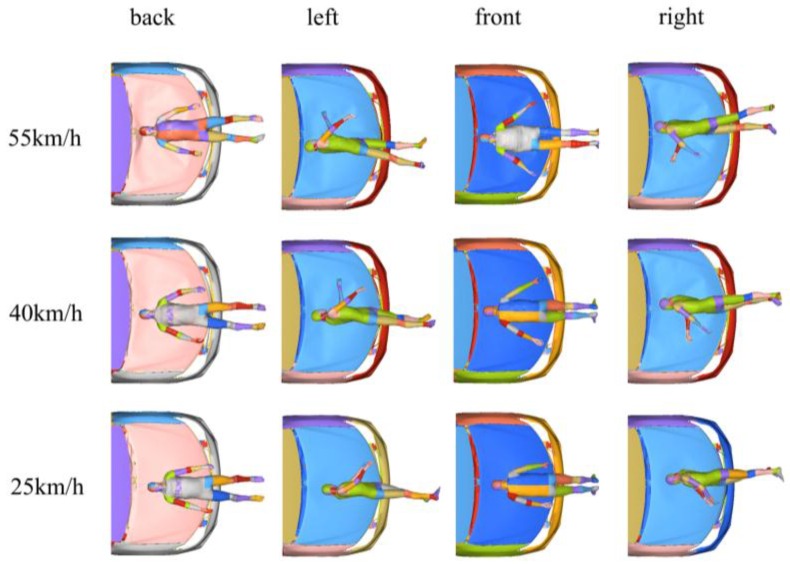
Head impact points at different collision parameters.

**Figure 5:**
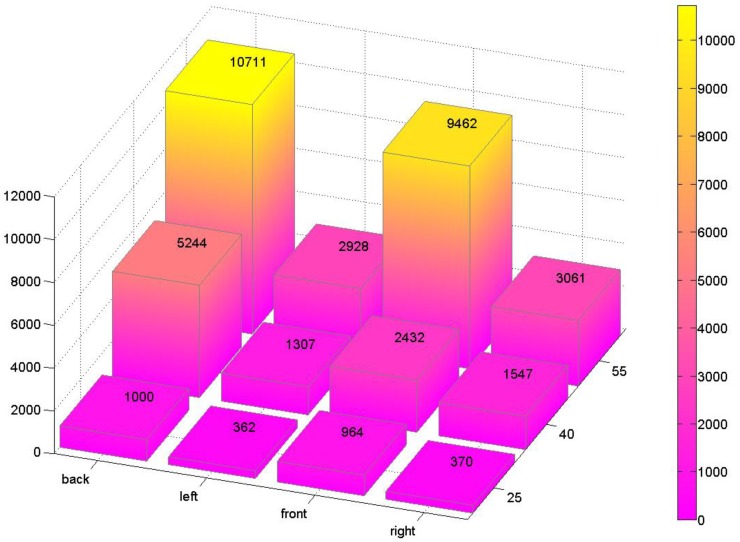
Head injury criterion (HIC) at different collision parameters.

When the car impacts the pedestrian at different velocities and angles, the head impact points and HIC of the pedestrian are shown in Figure 4 and Figure 5, respectively. It could be observed that the HIC values varied depending on the collision velocity and impact direction. At the impact velocities of 25, 40, and 55 km/h, the maximum values of the HIC were respectively 1000 (back impact), 5244 (back impact), and 10,711 (back impact). With an increase in the collision velocity, HIC increased; when the collision velocity remained constant, the HIC of the pedestrian under a back impact was successively greater than that impacted on the front and on the side.

Furthermore, a high HIC value was found for 55 km/h. One likely factor was that the head impact location was close to the rear edge of the hood. Another probable factor was that the pedestrian head injury severity changed with differences in physical status. A down-scaled pedestrian model could suffer a higher HIC value.

#### 3.2.2. Skull Stress

**Figure 6:**
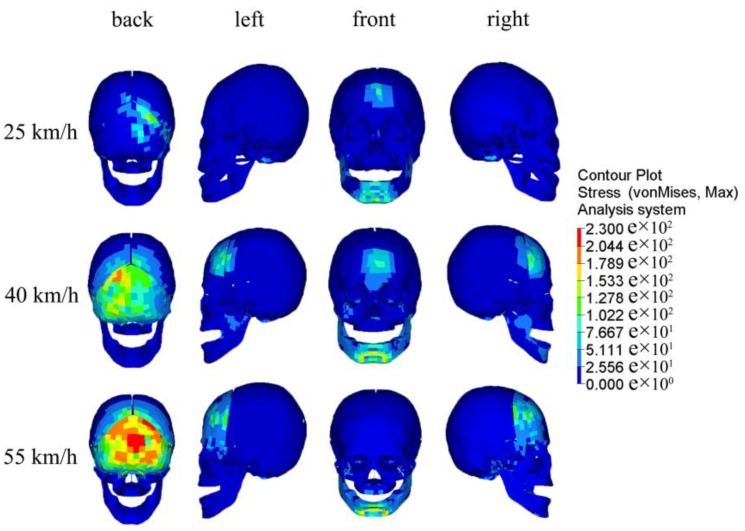
The Von Mises stress cloud on the skull at different collision parameters.

**Figure 7:**
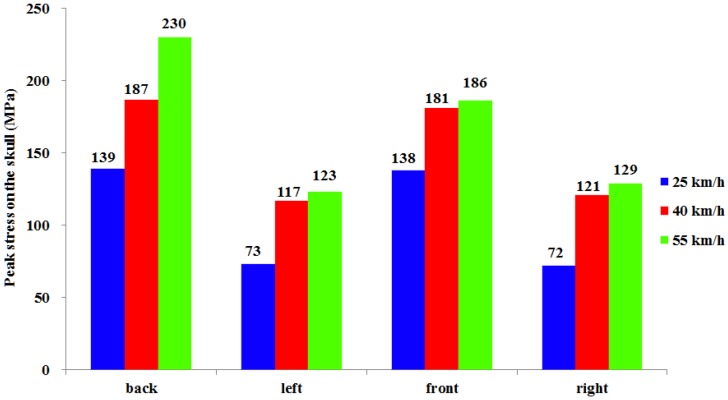
Peak stress on the skull at different collision parameters.

When a car impacts a pedestrian at different velocities and angles, with peak stress on the skull, the Von Mises stress cloud varies as shown in Figure 6. Simulation results showed that, at the moment of the head impact, a stress concentration was first formed at the impact region; then, stress gradually spread from the contact point to the surrounding areas; and finally, continuous reduction occurs until it eventually runs out of collision energy. At the same contact angle with different impact velocities, stress waves presented a similar mode of transmission on the skull, but peak stress values were different. When impact velocities were 25, 40, and 55 km/h, respectively, the maximum values of peak stress on the skull were 139 (back impact), 187 (back impact), and 230 MPa (back impact); and at the same velocities, peak stress values on the skull from the impact on the pedestrian’s back were successively greater than on the front and the side (see Figure 7).

#### 3.2.3. Brain Strain

**Figure 8:**
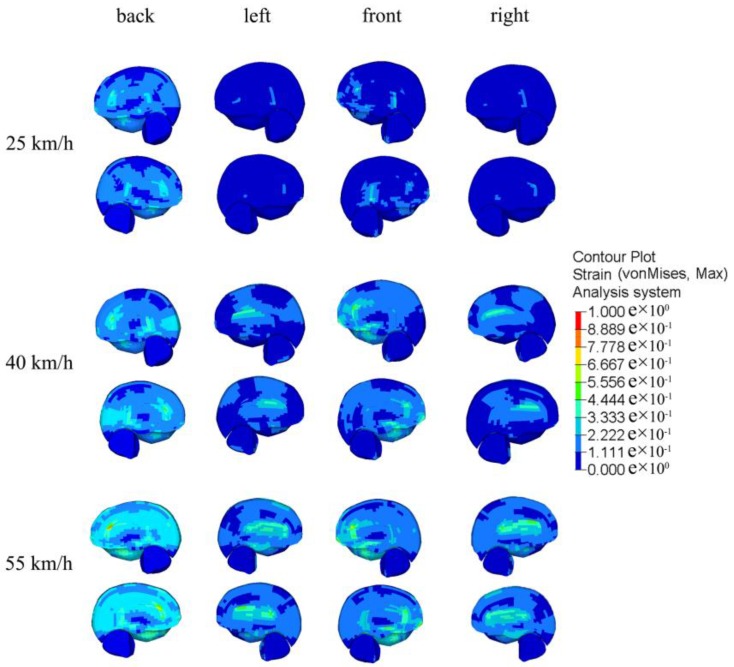
Parasagittal view of brain strain at different collision parameters.

**Figure 9:**
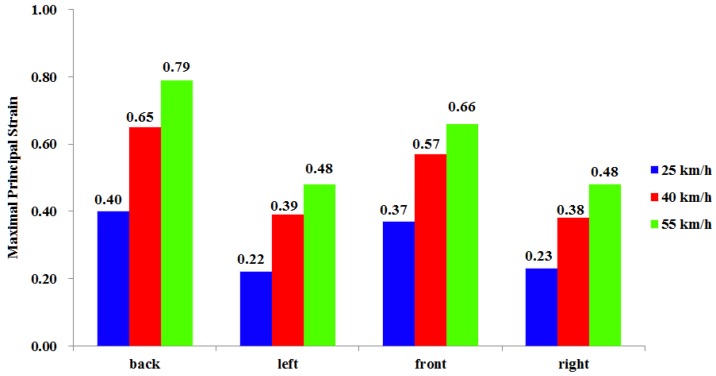
Maximal principal strain at different collision parameters.

Figure 8 illustrates the brain strain when a car impacts a pedestrian at different velocities and angles. At the same contact angle with different impact velocities, presented brain strain showed similar responses, but maximal principal strains for the brain were different. When impact velocities were 25, 40 and 55 km/h, respectively, the maximal principal strain for the brain were 0.40 (back impact), 0.65 (back impact), and 0.79 (back impact); at the same velocity, the maximal principal strain for the brain from the impact on the pedestrian’s back were successively greater than on the front and the side (see Figure 9).

#### 3.2.4. Injury Reconstruction and Prediction

A summary of the results from the reconstruction of the two different pedestrian head–hood impacts using the scaled THUMS is shown in Table 4. The head injury severity, skull fracture, and traumatic brain injuries were compared against the reconstruction results. Head injury reconstruction and prediction results were consistent with the real injuries. Fatal head injuries occurred in both cases. Although impact velocities have no obvious difference, Case 2, in which the pedestrian suffered a back impact, showed higher values of HIC, peak stress on the skull, and maximal principal strain for the brain.

## 4. Discussion

Previous studies have found that the head was one of the three most commonly injured parts of pedestrians in car–pedestrian crashes [9,43]. Serious head injuries can be life threatening and/or cause unpredictable consequences. Nowadays, there are many related materials reported in the research of pedestrian head injuries [19,20,21,22,23,24,25,26,27,28,29,30,43]. In addition, the application of HIC [49], maximum positive pressure, and minimum negative pressure [5] in head injuries assessment has been investigated. However, studies on the relationship between pedestrian head injuries and collision parameters are still in its infancy.

In car–pedestrian collisions, the biomechanical responses of the pedestrian’s head damages from impact are mainly compression and deformation of bone structure and soft tissue. The bone structure and soft tissue are deformed by pedestrian head injuries under compression load, sticky load, or organ inertia load, and when deformation exceeds the corresponding tolerance limit, skull fractures and brain contusions and lacerations occur, and so on [5,49]. From the basis of head injury information from real car–pedestrian accidents, a retrospective analysis of pedestrian head injuries was performed in this study. Furthermore, FEM was adopted for car–pedestrian crash simulation analysis. A combined analysis of results presented in Figure 5, Figure 6, Figure 7, Figure 8 and Figure 9 shows that the higher the car–pedestrian collision velocity was, the more serious the corresponding head injuries would be. This finding was in agreement with the results shown in Table 3 that the higher the collision velocity, the higher the proportion of skull fractures, brain contusions and lacerations, and head injuries at AIS3+; while at the same collision velocity, with the contact location of the car–pedestrian approximately the same, when a pedestrian was impacted on the back, the risk of skull and brain injury was greater than that from a side impact. This was consistent with the analysis results in Table 4 that the proportion of bilateral skull fractures and contusion and laceration of brain under a back collision was higher than under a side collision.

Consistent with some previously reported results [16,17,18,19], increased head impact velocity results in greater head injury severity. The analysis of HIC, peak stress on the skull, and maximal principal strain for the brain pointed to the same conclusion. As research has shown, increased collision velocity caused a greater value of HIC, peak stress on the skull, and maximal principal strain for the brain. With impact velocities of 25, 40, and 55 km/h, according to the probability curve of skull fracture established by Hertz [50] and HIC from collision simulation, the theoretical probability of skull fracture in car–pedestrian collisions was approximately 10%, 70% and 90%, respectively. The retrospective analysis found that in 25–39, 40–55, and ≥55 km/h, the proportion of pedestrian skull fractures were 3/12 (25%), 15/21 (71%), and 25/28 (89%), respectively. This also verified that the head injury simulation analysis and the statistical results were in good agreement.

Head injury simulation analysis clearly pointed out the relationship between impact velocity and brain deformations. It was observed that increased head impact velocity results in greater maximal principal strain on the brain. A similar finding was also reported by Mordaka [11], who found a dramatic, three-fold increase in the strain levels in the brain when the impact velocity was doubled.

Head injury reconstruction and prediction results of the two selected cases were well consistent with the real injuries. The simulation results can be used to predict real-world head injuries caused by head–hood impact for shorter stature pedestrians. Although impact velocities have no obvious difference in both cases, Case 2, in which the pedestrian suffered a back impact, showed higher values of HIC, peak stress on the skull and maximal principal strain for the brain. The reconstruction data also revealed that head injury severity was not only related to impact velocity, but also related to the contact angle.

Although the collision simulation analysis of the back and the side impacts matched with the characteristics of head injuries, there was still a lack of crashes under a front impact in the retrospective analysis, which should be addressed in future work. Two other aspects that need to be looked at in the future studies are individual-level characteristics of pedestrians including age, sex, height, and weight, and pedestrian head movement characteristics such as transverse translation and head rotation. Age has been hypothesized to be a significant determinant of pedestrian injury severity with older adults being at greater risk than younger pedestrians [51]. Additionally, transverse translation, head rotation, and head impact velocity have been found to vary cyclically with gait in clearly definable characteristics [19].

## 5. Conclusions

The present study is intended to discuss head injury characteristics in car–pedestrian collisions. Through a combination of retrospective analyses and finite element method analyses, statistical data and damage assessment parameters have been quantitatively calculated to explore the relationship between pedestrian head injuries and car–pedestrian collision parameters. It is verified that the collision simulation analysis has matched with the statistical results, and the study has demonstrated, once again, increased head impact velocity results in greater head injury severity. Furthermore, head injury reconstruction and prediction results of two selected cases were consistent with real injuries. For shorter stature pedestrians, the head subjected to rear impact has been found to be at greater damage risk, which may have implications on automotive design and pedestrian protection research if prevention and treatment of these injuries is to be prioritized over injuries of heads subjected to front or side impact.

## Figures and Tables

**Table 1:** Initial conditions for cases.

	Age, Gender	Impact Velocity, Contact Angle	Injury Description	Impacted Structure
Case 1	59, female	51 km/h, left	Superficial scalp injury (5.0 cm × 4.0 cm, right side), Scalp hematoma in temporal and parietal area (8.0 cm × 8.0 cm, left side), Subdural hematomas in temporal area (left side), Skull fracture (left side)	Car engine hood
Case 2	50, female	55 km/h, back	Superficial scalp injury (5.5 cm × 4.4 cm, back side), Scalp hematoma in temporal area (2.4 cm × 1.5 cm, left side), Foramen magnum puncture site bleeding, Skull fracture (back side)	Car engine hood

**Table 2:** Comparison of head injuries at different collision velocities.

Velocities (km/h)	Contusion and Laceration of Scalp ^1^			Skull Fractures ^2^			Contusion and Laceration of Brain ^3^			Head Injuries AIS ^4^		
	No Contusion or Laceration	Unilateral	Bilateral	No Fracture	Unilateral	Bilateral	No Contusion or Laceration	Unilateral	Bilateral	(6, 5)	(4, 3)	(2, 1)
25–39	3	8	1	9	2	1	11	1	0	0	7	5
40–55	7	11	3	6	11	4	19	2	0	2	17	2
>55	10	14	4	3	19	6	14	6	8	12	16	0

^1^ *p* = 0.943; ^2^ *p* = 0.003; ^3^ *p* = 0.005; ^4^ *p* < 0.001.

**Table 3:** Comparison of head injuries at different contact angles.

Contact Angles	Contusion and Laceration of Scalp ^1^				Skull Fractures ^2^				Contusion and Laceration of Brain ^3^				Head Injuries AIS ^4^		
	No Contusion or Laceration	Left	Right	Bilateral	No Fracture	Left	Right	Bilateral	No Contusion or Laceration	Left	Right	Bilateral	(6, 5)	(4, 3)	(2, 1)
Left	10	2	18	3	9	19	1	4	25	5	0	3	3	25	5
Right	8	8	4	1	7	0	11	3	15	0	3	3	12	7	2
Back	2	0	1	4	2	0	1	4	4	0	1	2	4	3	0

^1^ *p* = 0.001; ^2^ *p* < 0.001; ^3^ *p* = 0.001; ^4^ *p* = 0.001.

**Table 4:** Injury reconstruction and prediction results of head.

	Real Injury			Injury Reconstruction			Injury Prediction		
	Head Injury Severity	Skull Fracture	Traumatic Brain Injuries	HIC	Peak Stress on the Skull	Maximal Principal Strain for the Brain	Head Injury Severity	Skull Fracture	Traumatic Brain Injuries
Case 1	fatal	yes	yes	1909	185 MPa	0.45	fatal	yes	yes
Case 2	fatal	yes	yes	10711	230 MPa	0.79	fatal	yes	yes

## References

[1] Simms C.K., Wood D.P. (2006). Effects of pre-impact pedestrian position and motion on kinematics and injuries from vehicle and ground contact. Int. J. Crashworth..

[2] Simms C.K., Wood D.P. (2006). Pedestrian risk from cars and sport utility vehicles—A comparative analytical study. Proc. Inst. Mech. Eng. Part D J. Automob. Eng..

[3] Untaroiu C.D., Meissner M.U., Crandall J.R., Takahashi Y., Okamoto M., Ito O. (2009). Crash reconstruction of pedestrian accidents using optimization techniques. Int. J. Impact Eng..

[4] Lan F.C., Cai Z.H., Chen J.Q., Ma Z.W. (2012). Biomechanical responses and injury evaluation of human thorax and abdomen during vehicle collision. J. South China Univ. Technol..

[5] Cao L.B., Zhou Z., Jiang B.H., Zhang G.J. (2014). Development and validation of the FE model for a 10-year-old child head. Chin. J. Biomed. Eng..

[6] Wei Z. (2011). Development of human head finite element model on biomechanical research. Chin. J. Biomed. Eng..

[7] Liu W.F., Wang F., Wang D.B., Chen X.J., Wang Y., Surgery D.O., Amp E., Group E. (2013). The present application of finite element method in craniocerebral injury. Med. Recapitul..

[8] Ruan S.J., He P., Lu J.P., Li H.Y., Li X., Zhang H.Z. (2007). Simulation based investigation on injury biomechanics of the human head by finite element method. Chin. J. Biomed. Eng..

[9] Liu W., Hui Z., Li K., Su S., Fan X., Yin Z. (2015). Study on pedestrian thorax injury in vehicle-to-pedestrian collisions using finite element analysis. Chin. J. Traumatol..

[10] Jingwen H.U., Jonathan D., Matthew P. (2012). Focusing on vulnerable populations in crashes: Recent advances in finite element human models for injury biomechanics research. J. Automot. Saf. Energy.

[11] Mordaka J., Kleiven S., Schijndel-De N.V., Lange R.D., Guerra C.L., Carter E., Holst H.V. The Importance of Rotational Kinematics in Pedestrian Head to Windshield Impacts. Proceedings of the 2007 International IRCOBI Conference on the Biomechanics of Injury.

[12] Watanabe R., Katsuhara T., Miyazaki H., Kitagawa Y., Yasuki T. (2012). Research of the relationship of pedestrian injury to collision speed, car-type, impact location and pedestrian sizes using human FE model (THUMS version 4). Stapp Car Crash J..

[13] Alvarez V.S., Halldin P., Kleiven S. (2014). The influence of neck muscle tonus and posture on brain tissue strain in pedestrian head impacts. Stapp Car Crash J..

[14] Fahlstedt M., Halldin P., Kleiven S. (2016). Comparison of multibody and finite element human body models in pedestrian accidents with the focus on head kinematics. Traffic Inj. Prev..

[15] Nilsson G. (2004). Traffic Safety Dimensions and the Power Model to Describe the Effect of Speed on Safety.

[16] Elvik R. (2013). A re-parameterisation of the power model of the relationship between the speed of traffic and the number of accidents and accident victims. Accid. Anal. Prev..

[17] Tefft B.C. (2013). Impact speed and a pedestrian's risk of severe injury or death. Accid. Anal. Prev..

[18] Jurewicz C., Sobhani A., Woolley J., Dutschke J., Corben B. Exploration of Vehicle Impact Speed—Injury Severity Relationships for Application in Safer Road Design. Proceedings of the Transport Research Arena.

[19] Elliott J.R., Simms C.K., Wood D.P. (2012). Pedestrian head translation, rotation and impact velocity: The influence of vehicle speed, pedestrian speed and pedestrian gait. Accid. Anal. Prev..

[20] Richards D., Carroll J. (2012). Relationship between types of head injury and age of pedestrian. Accid. Anal. Prev..

[21] Hu J., Klinich K.D. (2012). Toward designing pedestrian-friendly vehicles. Int. J. Veh. Saf..

[22] Mizuno Y., Ishikawa H. Summary of IHRA Pedestrian Safety Working Group Activities Proposed Test Methods to Evaluate Pedestrian Protection Afforded by Passenger Cars. Proceedings of the 19th International Technical Conference on the Enhanced Safety of Vehicles (ESV).

[23] Fredriksson R., Zhang L., Boström O., Yang K. (2007). Influence of impact speed on head and brain injury outcome in vulnerable road user impacts to the car hood. Stapp Car Crash J..

[24] Zhang G., Cao L., Hu J., Yang K.H. (2008). A field data analysis of risk factors affecting the injury risks in vehicle-to-pedestrian crashes. Ann. Adv. Automot. Med..

[25] McLean J., Robinson G.K. (1979). Adelaide In-Depth Accident Study 1975–1979. Part 1: An Overview.

[26] Alshammari N., Bendak S., Algadhi S. (2009). In-depth analysis of pedestrian crashes in Riyadh. Traff. Inj. Prev..

[27] Kong C., Yang J. (2010). Logistic regression analysis of pedestrian casualty risk in passenger vehicle collisions in China. Accid. Anal. Prev..

[28] Zhao H., Yin Z., Chen R., Chen H., Song C., Yang G., Wang Z. (2010). Investigation of 184 passenger car–pedestrian accidents. Int. J. Crashworth..

[29] Chen H., Fu L., Zheng H. (2009). A comparative study between China and IHRA for the vehicle–pedestrian impact. SAE Int. J. Passeng. Cars Mech. Syst..

[30] Zheng H., Liu J.Y., Song F.J., Chen K.X. (2013). An investigation on the head injuries of adult pedestrians by passenger cars in China. Traff. Inj. Prev..

[31] Civil I.D., Schwab C.W. (1988). The abbreviated injury scale, 1985 revision: A condensed chart for clinical use. J. Trauma.

[32] Zou T., Yu Z., Cai M., Liu J. (2011). Analysis and application of relationship between post-braking-distance and throw distance in vehicle–pedestrian accident reconstruction. Forensic Sci. Int..

[33] Xu S., Yang S., Chen C., Wu G. Speed Identification Based on Surveillance Video in Traffic Accidents. Proceedings of the International Conference on Intelligent Control and Computer Application (ICCA 2016).

[34] Su S., Liu W.J., Wang F.P., Li K., Yin Z.Y. (2014). Comparison of different means to determine postcrash velocity in a real-world vehicle–pedestrian accident. Appl. Mech. Mater..

[35] Thole C.A. Advanced Mode Analysis for Crash Simulation Results. Proceedings of the 11th International LS-DYNA Conference.

[36] Iza-Teran R. (2014). Enabling the analysis of finite element simulation bundles. Int. J. Uncertain. Quantif..

[37] Hou S., Dong D., Ren L., Han X. (2012). Multivariable crashworthiness optimization of vehicle body by unreplicated saturated factorial design. Struct. Multidiscip. Optim..

[38] NCAC. http://www.ncac.gwu.edu/vml/models.html.

[39] Marzougui D., Brown D., Park H.K., Kan C.D., Opiela K.S. Development & Validation of a Finite Element Model for a Mid-Sized Passenger Sedan. Proceedings of the 13th International LS-DYNA Users Conference.

[40] TMC (2011). THUMS User Manual, am50 Pedestrian/Occupant Model, Academic Version 4.0_20111003.

[41] Watanabe R., Miyazaki H., Kitagawa Y., Yasuki T. (2012). Research of collision speed dependency of pedestrian head and chest injuries using human FE model (THUMS version 4). Accid. Reconstr. J..

[42] Total Human Model for Safety—THUMS. http://www.lstc.com/thums.

[43] Yang J. (2011). Overview of research on injury biomechanics in car–pedestrian collisions. Chin. J. Automot. Eng..

[44] Paas R., Östh J., Davidsson J. Which Pragmatic Finite Element Human Body Model Scaling Technique Can Most Accurately Predict Head Impact Conditions in Pedestrian–Car Crashes?. Proceedings of the 2015 IRCOBI conference Proceedings.

[45] Mackay M. (2007). The increasing importance of the biomechanics of impact trauma. Sadhana.

[46] Mcelhaney J.H., Fogle J.L., Melvin J.W., Haynes R.R., Roberts V.L., Alem N.M. (1970). Mechanical properties on cranial bone. J. Biomech..

[47] Thibault L.E., Gennarelli T.A., Margulies S.S., Marcus J., Eppinger R. The Strain Dependent Pathophysiological Consequences of Inertial Loading on Central Nervous System tissue. Proceedings of the International IRCOBI Conference on the Biomechanics of Impacts.

[48] Rd M.B., Cater H.L., Wang C.C., Thomas F.C., Hung C.T., Ateshian G.A., Sundstrom L.E. (2003). A tissue level tolerance criterion for living brain developed with an in vitro model of traumatic mechanical loading. Stapp Car Crash J..

[49] Shijie R. (2007). A new exploration of the applicability of the head injury criterion. J. Biomed. Eng..

[50] Hertz E. A Note on the Head Injury Criterion (HIC) as a Predictor of the Risk of Skull Fracture. Proceedings of the 37th Annual Conference Association for the Advancement of Automotive Medicine.

[51] Niebuhr T., Junge M., Rosén E. (2016). Pedestrian injury risk and the effect of age. Accid. Anal. Prev..

